# ASSESSMENT OF CELL SURFACE TARGETS IN METASTATIC PROSTATE CANCER: EXPRESSION LANDSCAPE AND MOLECULAR CORRELATES

**DOI:** 10.21203/rs.3.rs-3745991/v1

**Published:** 2023-12-19

**Authors:** Azra Ajkunic, Erolcan Sayar, Martine P. Roudier, Radhika A. Patel, Ilsa M. Coleman, Navonil De Sarkar, Brian Hanratty, Mohamed Adil, Jimmy Zhao, Samir Zaidi, Lawrence D. True, Jamie M. Sperger, Heather H. Cheng, Evan Y. Yu, Robert B. Montgomery, Jessica E. Hawley, Gavin Ha, John K. Lee, Stephanie A. Harmon, Eva Corey, Joshua M. Lang, Charles L. Sawyers, Colm Morrissey, Michael T. Schweizer, Roman Gulati, Peter S. Nelson, Michael C. Haffner

**Affiliations:** 1 Division of Human Biology, Fred Hutchinson Cancer Center, Seattle, WA, USA; 2 Department of Urology, University of Washington, Seattle, WA, USA; 3 Medical College of Wisconsin Cancer Center, Milwaukee, WI, USA; 4 Department of Pathology, Medical College of Wisconsin, WI, USA; 5 Human Oncology and Pathogenesis Program, Memorial Sloan Kettering Cancer Center, New York, NY, USA; 6 Department of Laboratory Medicine and Pathology, University of Washington, Seattle, WA, USA; 7 University of Wisconsin-Madison, Madison, WI, USA; 8 Division of Clinical Research, Fred Hutchinson Cancer Center, Seattle, WA, USA; 9 Division of Hematology and Oncology, Department of Medicine, University of Washington, Seattle, WA, USA; 10 Public Health Sciences Division, Fred Hutchinson Cancer Center, Seattle, WA, USA; 11 Department of Genome Sciences, University of Washington, Seattle, WA, USA.; 12 Artificial Intelligence Resource, Molecular Imaging Branch, NCI, NIH, Bethesda, MD, USA; 13 Howard Hughes Medical Institute, Memorial Sloan Kettering Cancer Center, New York, NY, USA

## Abstract

Therapeutic approaches targeting proteins on the surface of cancer cells have emerged as an important strategy for precision oncology. To fully capitalize on the potential impact of drugs targeting surface proteins, detailed knowledge about the expression patterns of the target proteins in tumor tissues is required. In castration-resistant prostate cancer (CRPC), agents targeting prostate-specific membrane antigen (PSMA) have demonstrated clinical activity. However, PSMA expression is lost in a significant number of CRPC tumors, and the identification of additional cell surface targets is necessary in order to develop new therapeutic approaches. Here, we performed a comprehensive analysis of the expression and co-expression patterns of trophoblast cell-surface antigen 2 (TROP2), delta-like ligand 3 (DLL3), and carcinoembryonic antigen-related cell adhesion molecule 5 (CEACAM5) in CRPC samples from a rapid autopsy cohort. We show that DLL3 and CEACAM5 exhibit the highest expression in neuroendocrine prostate cancer (NEPC), while TROP2 is expressed across different CRPC molecular subtypes, except for NEPC. We observed variable intra-tumoral and inter-tumoral heterogeneity and no dominant metastatic site predilections for TROP2, DLL3, and CEACAM5. We further show that *AR* amplifications were associated with higher expression of PSMA and TROP2 but lower DLL3 and CEACAM5 levels. Conversely, PSMA and TROP2 expression was lower in *RB1*-altered tumors. In addition to genomic alterations, we demonstrate a tight correlation between epigenetic states, particularly histone H3 lysine 27 methylation (H3K27me3) at the transcriptional start site and gene body of *TACSTD2 (encoding TROP2)*, *DLL3*, and *CEACAM5*, and their respective protein expression in CRPC patient-derived xenografts. Collectively, these findings provide novel insights into the patterns and determinants of expression of TROP2, DLL3, and CEACAM5 with important implications for the clinical development of cell surface targeting agents in CRPC.

## INTRODUCTION

Prostate cancer (**PC**) ranks as the second leading cause of cancer-related deaths among American men, claiming over 34,700 lives annually ^1^. While androgen deprivation therapy (**ADT**) is initially effective in most men with advanced PC, the emergence of castration-resistant prostate cancer (**CRPC**) and resistance to androgen receptor (**AR)** signaling inhibitors (**ARSIs**) develops in almost all patients ^2,3^.

It is increasingly recognized that resistance to contemporary AR targeting therapies is associated with a diverse spectrum of disease phenotypes characterized by morphologic and molecular changes, which are often associated with a loss of prostate lineage features (such as the expression of AR) and the gain of more stem-like and neuronal features ^4–7^. Therefore, disease subclassifications were proposed that are based on the assessment of AR and neuroendocrine marker expression ^5,8,9^. Among these molecular subtypes, neuroendocrine prostate cancer (**NEPC**), characterized by the absence of AR signaling and gain of neuroendocrine features, represents the most aggressive disease subtype, with chemotherapy as the only available treatment option ^4,10,11^. There is, therefore, a critical clinical need for novel therapeutics in this difficult-to-treat and prognostically poor subset of patients.

Targeting cell-surface antigens through the delivery of cytotoxic agents directly to cancer sites or by generating anti-tumor immune responses are promising therapeutic approaches for advanced cancers ^12–17^. Prostate-specific membrane antigen (**PSMA**) is currently the most extensively validated theranostic cell surface target in PC ^18,19^. Although PSMA shows a favorable and relatively prostate lineage-restricted expression, up to 40% of CRPC patients show loss or heterogeneous PSMA expression ^18,20–22^. In particular, the absence of PSMA expression is nearly universal in NEPC ^20,21^. To maximize the therapeutic benefit, there is a great need to understand the expression patterns of other cell surface proteins.

Of the constantly expanding spectrum of cell-surface targets in oncology, delta-like ligand 3 (**DLL3**), carcinoembryonic antigen-related cell adhesion molecule 5 (**CEACAM5**), and trophoblast cell-surface antigen 2 (**TROP2**) have been a focus for pre-clinical and clinical drug development efforts for advanced PC ^23–28^.

DLL3 is a ligand that inhibits the Notch signaling pathway and is expressed in the spinal cord and nervous system during embryonic development ^24^. Importantly, DLL3 is expressed at high levels in the majority of tumors that exhibit high-grade neuroendocrine/small cell carcinoma features, making it a potentially valuable target for NEPC ^23–25,29^. Similarly, CEACAM5, a member of the carcinoembryonic antigen family, is overexpressed in a larger fraction of solid tumors, with high expression observed in NEPC ^26,30^. Notably, several antibody-drug conjugates (**ADCs**) targeting CEACAM5 have been developed and explored in the context of different solid tumors ^26,31,32^. TROP2 is a transmembrane protein that is expressed in multiple malignancies^27,33–36^. Clinical trials using TROP2-targeting agents have shown efficacy and a TROP2 ADC sacituzumab govitecan has been approved for triple-negative breast cancer and urothelial carcinoma, and phase 2 studies in CRPC are currently ongoing ^35,36^.

Since the efficacy of DLL3-, CEACAM5-, and TROP2-targeting strategies will in part depend on the expression of these antigens, it is informative to examine their expression in clinically relevant and well-annotated metastatic CRPC (**mCRPC**) cohorts. From a clinical perspective, it is particularly relevant to understand antigen expression across different molecular subtypes of PC and to establish the inter- and intra-patient expression variability. While prior studies have established the expression of these proteins in smaller PC cohorts, their patterns of expression and intra- and inter-tumoral heterogeneity have not been rigorously studied in metastatic CRPC. This is largely due to the difficulties of accessing biospecimen cohorts across diverse metastatic sites that provide a comprehensive representation of the metastatic tumor burden within and across different patients ^7^.

In this study, we determined the expression of DLL3, CEACAM5 and TROP2 in 753 tissue samples from 52 mCRPC patients. Leveraging the unique biospecimens from the University of Washington rapid autopsy cohort, we show that DLL3 and CEACAM5 expression is mostly restricted to tumors lacking AR signaling activity and expressing neuroendocrine markers. Conversely, TROP2 is expressed at high levels in most tumors except for NEPC. Despite these molecular subtype-specific expression differences, we demonstrate that TROP2 and DLL3 show relatively limited inter-tumoral heterogeneity. In addition, we show a relative enrichment of cell surface antigen expression in certain somatic genomic backgrounds, and we highlight a novel epigenetic mechanism involved in the regulation of DLL3, CEACAM5, and TROP2. These data provide valuable information on therapeutic target expression in CRPC and present a rationale for informed co-targeting strategies.

## RESULTS

### Patterns of DLL3, CEACAM5, PSMA and TROP2 protein expression across molecular subtypes of mCRPC

To contextualize the expression patterns of cell surface antigens in mCRPC, we utilized a recently published molecular subgrouping framework based on AR signaling and neuroendocrine (**NE**) marker expression ^5,6,20,37^. This approach allows for the classification of tumors into four clinically relevant subtypes: prostatic adenocarcinoma (AR+/NE−), NEPC (AR−/NE+), amphicrine carcinoma (AR+/NE+) and double negative CRPC (AR−/NE−) ^20,37^. To investigate the expression of DLL3, CEACAM5, and TROP2, we employed previously validated antibodies and immunohistochemistry (**IHC**) assays on a dataset consisting of 753 samples from 372 distinct metastatic sites of 52 patients who underwent a rapid autopsy as part of the University of Washington Tissue Acquisition Necropsy (**UW-TAN**) cohort ^5,6^.

**Figure 1.**
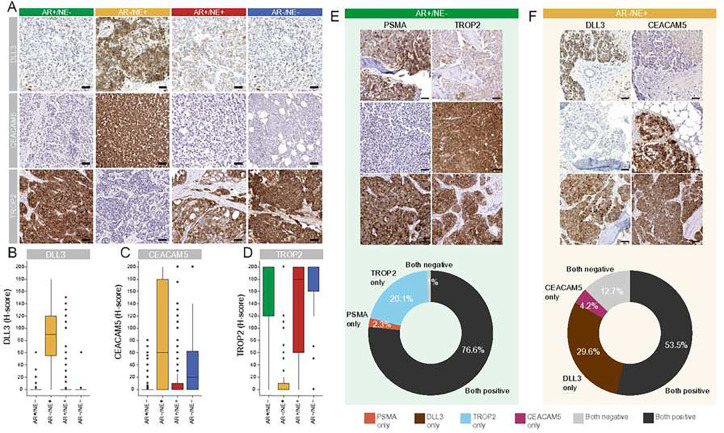
Distribution and co-expression patterns of DLL3, CEACAM5, PSMA, and TROP2 expressions across different molecular subtypes of mCRPC. **A.** Representative images of cell surface antigen expressions (determined by IHC) across different molecular subtypes (AR+/NE− [green], AR−/NE+ [yellow], AR+/NE+ [red], and AR−/NE− [blue]). Molecular subtypes were defined by expression of AR signaling markers (AR, NKX3.1) and NE markers (SYP, INSM1) as described previously ^20^. Box plots show the distribution of **B.** DLL3, **C.** CEACAM5, and **D.** TROP2 expressions based on H-score in the UW-TAN cohort (N=753). Box and dot colors indicate molecular phenotypes as above. **E.** Top, micrographs of PSMA and TROP2 in AR+/NE− tumors. Bottom, donut chart shows the distribution of PSMA and TROP2 reactivity. **F.** Top, micrographs of DLL3 and CEACAM5 in AR−/NE+ tumors. Bottom, donut chart shows the distribution of DLL3 and CEACAM5 reactivity. (See **Supplementary Table 2** for all co-expression profiles). Scale bars denote 50 μm.

DLL3, CEACAM5, and TROP2 exhibited membranous and cytoplasmic reactivity, with substantial differences in semiquantitative expression levels (H-score) across different molecular subtypes (Figure 1A). Consistent with prior reports, we observed the highest levels of DLL3 expression in AR−/NE+ tumors (median H-score: 90; range, 0–180) (Figure 1A,B) ^24,25^. Similarly, CEACAM5 expression was high in AR−/NE+ tumors (median, 60; range, 0–200) (Figure 1A,C) ^26^. Of note, we also observed CEACAM5 reactivity in AR−/NE− tumors (Figure 1C). TROP2 expression was consistently present in AR+/NE− (median H-score: 200; range, 0–200), AR+/NE+ (median H-score: 180; range, 0–200), and AR−/NE− (median H-score: 200; range, 0–200) tumors (Figure 1A,D), whereas AR−/NE+ tumors were mostly negative (median, 0; range, 0–200). Notably, TROP2 showed more uniform expression compared to PSMA in the same cohort of AR+/NE− (median H-score: 120; range, 0–200) and AR−/NE− (median H-score: 12; range, 0–160) tumors ^20^.

Next, we determined the co-expression patterns of cell surface antigens across patients. Applying a cut-off for positive expression of an H-score of ≥20 (**Supplementary Figure 1**), we found that in AR+/NE− tumors 233/304 (77%) of lesions showed expression of both TROP2 and PSMA, 7/304 (2%) were positive only for PSMA, 61/304 (20%) were positive only for TROP2 and 3/304 (1%) showed neither PSMA nor TROP2 (Figure 1E, **Supplementary Table 2**, **Supplementary Figure 2**). Similarly, in AR−/NE+ tumors, we found DLL3 and CEACAM5 co-expression in 38/71 (54%) tumors, DLL3 expression alone in 21/71 (30%), CEACAM5 expression alone in 3/71 (4.2%) and expression of neither target in 9/71 (13%) (Figure 1F, **Supplementary Table 2**, **Supplementary Figure 2**).

### Anatomic site distribution and inter- and intra-tumoral heterogeneity of TROP2, DLL3, and CEACAM5 expression

**Figure 2.**
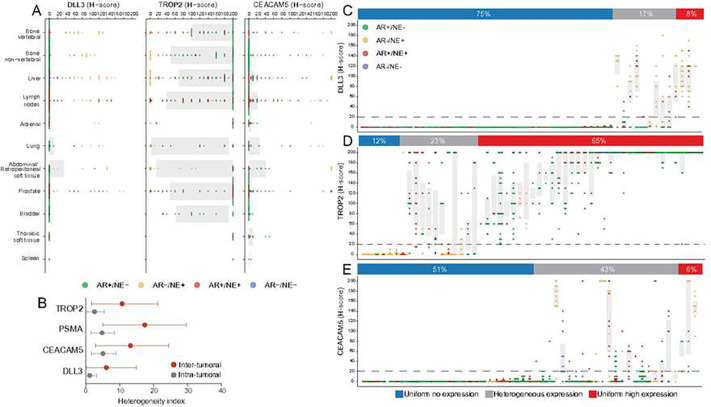
Anatomic site distribution and inter- and intra-tumoral heterogeneity of TROP2, DLL3 and CEACAM5 expression in mCRPC. **A.** Distribution of DLL3, TROP2, and CEACAM5 protein expression across different organ sites based on IHC H-scores. Dot colors indicate molecular phenotypes. Each dot represents a tumor sample; the color codes indicate the molecular subtype (AR+/NE− [green], AR−/NE+ [yellow], AR+/NE+ [red], and AR−/NE− [blue]). **B.** Inter- and intra-tumoral heterogeneity of TROP2, PSMA, CEACAM5 and DLL3 expression. Mean (95% confidence interval) hypergeometric expression heterogeneity indices across different metastatic sites in a given patient (inter-tumoral heterogeneity, red) and within a metastatic site (intra-tumoral heterogeneity, gray). Dot and box plots showing the distribution of **C.** DLL3, **D.** TROP2, and **E.** CEACAM5 protein expression IHC H-scores in 52 cases from the UW-TAN cohort. Each dot represents a tumor sample; the color codes indicate the molecular subtype (AR+/NE− [green], AR−/NE+ [yellow], AR+/NE+ [red], and AR−/NE− [blue]). Gray shadings show interquartile ranges. Percentages show the frequencies of cell surface antigens in cases with uniformly low/negative expression (all sites H-score <20), heterogeneous expression (both H-scores <20 and H-score ≥20) and uniformly high expression (all sites H-scores ≥20).

Prior studies suggested differences in cell surface protein expression based on the tumor microenvironment in different anatomic locations ^21^. Indeed, lower levels of PSMA expression were observed in liver metastases ^20,21^. To examine the association between anatomic location and the level of cell surface antigen expression, we assessed DLL3, CEACAM5, and TROP2 expression across 11 major anatomic sites of CRPC metastases (Figure 2A). While bone was the most common metastatic site in this cohort, we observed a high frequency of liver and soft tissue metastases, irrespective of the molecular tumor phenotype (Figure 2A). We observed significantly lower TROP2 expression in liver (mean H-score difference: −17; 95% CI −31 to −3.0; p=0.02) and lung (mean H-score difference: −40; 95% CI −65 to −15; p=0.001) than in vertebral bone metastases (mean H-score: 131; 95% CI 110 to 151). CEACAM5 expression in the prostate was significantly higher (mean H-score difference: 19; 95% CI 9.2 to 28; p<0.001) than in vertebral bone (mean H-score:19; 95% CI 5.6 to 33), whereas DLL3 expression was higher in liver (mean H-score difference: 11; 95% CI 5.5 to 17; p<0.001) and lung (mean H-score difference: 14; 95% CI 4.3 to 23; p=0.005) compared to vertebral bone metastases (mean H-score: 12; 95% CI 1.4 to 22). Note, while these differences were statistically significant, estimated differences in mean H-scores were very modest in magnitude and unlikely to be biologically relevant (Figure 2A).

CRPC is known to be a heterogeneous disease, often showing phenotypic differences between different metastatic sites in a given patient ^7,20,38^. To characterize the heterogeneity of TROP2, DLL3, and CEACAM5 expression, we quantified the hypergeometric probability of concordant binarized H-scores (both ≥20 or both <20) for random pairs of samples from a given patient (intra-patient, inter-tumoral) or from the same tumor (intra-tumoral) (Figure 2B). Estimated heterogeneity was highest for PSMA (intra-patient 17% and intra-tumoral 5%, previously reported^20^), then CEACAM5 (14% and 6%), then TROP2 (8% and 2%), and finally DLL3 (7% and 2%).

We analyzed TROP2, DLL3, and CEACAM5 expression levels across different metastatic sites and classified patients into three groups: non-expressors, heterogeneous expressors, and high-expressors. In 39/52 (75%) of cases, DLL3 showed no expression, while in 9/52 (17%) of cases, it showed heterogenous expression and in 4/52 (8%) of cases, it showed homogeneous high expression. Of note, most cases with heterogeneous expression displayed different molecular subtypes across metastatic sites. Furthermore, except for two cases (Figure 2C), DLL3 labeling was present in AR−/NE+ metastases, even in the context of metastases of other molecular subtypes, confirming the tight association between DLL3 expression and neuroendocrine differentiation even in admixed molecular phenotype backgrounds. TROP2 showed the most consistent expression among the three analytes tested in this study. Only 6/52 (12%) cases showed no expression, and negative cases were enriched for NE+ tumors, with only one AR+/NE− dominant case lacking TROP2 reactivity (Figure 2D). Heterogenous TROP2 expression was present in at least one tumor in 12/52 (23%) cases, while tumors in 34/52 cases (65%) were uniformly positive. This high rate of TROP2 expression compares favorably to the expression of PSMA in the same cohort (25% no expression, 44% heterogeneous, and 31% uniformly positive) ^20^. CEACAM5, on the other hand, was not expressed in 26/52 (51%) of cases, heterogeneously expressed in 22/51 (43%) of cases, and uniformly positive in only 3/51 (6%) of cases (Figure 2E). Notably, even in some cases which showed AR−/NE+ disease in the majority of metastases, CEACAM5 expression was low; conversely, a subset of tumors that lacked neuroendocrine features showed reactivity, suggesting that molecular subtype alone might not be sufficient to determine CEACAM5 expression.

### Genomic and epigenetic determinants of TROP2, PSMA, DLL3, and CEACAM5 expression

**Figure 3.**
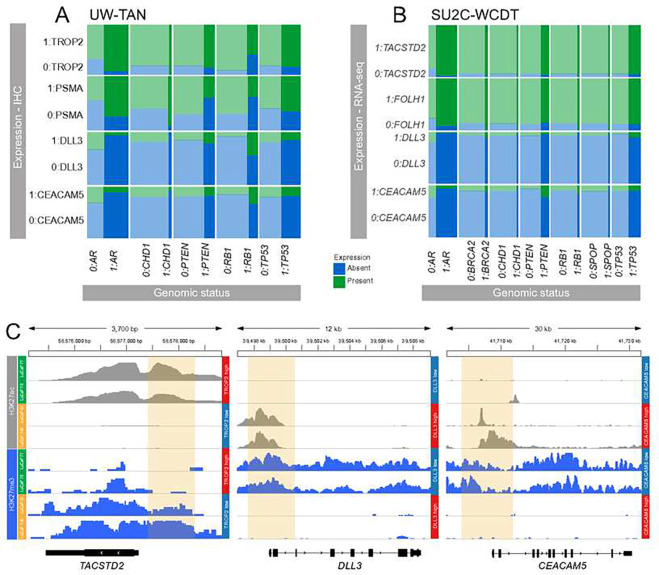
Genetic and epigenetic determinants of TROP2, PSMA, DLL3 and CEACAM5 expression in CRPC. **A.** Mosaic plots show the frequencies of TROP2, PSMA, DLL3, and CEACAM5 protein expression determined by IHC (1, expressed; 0, not expressed) as a function of the genomic status of *AR*, *CHD1*, *PTEN*, *RB1*, and *TP53* (1, altered; 0, not altered) in 44 cases of the UW-TAN cohort. **B.** Mosaic plots show the frequencies of *TACSTD2*, *FOLH1*, *DLL3* and CEACAM5 mRNA expression determined by RNA-seq (1, expressed; 0, not expressed) as a function of the genomic status of *AR*, *BRCA2*, *CHD1*, *PTEN*, *RB1, SPOP*, and *TP53* (1, altered; 0, not altered) in 99 cases of the SU2C-WCDT. **C.** Representative H3K27ac (gray) and H3K27me3 ChIP-seq tracks from AR+/NE− (LuCaP 77 and LuCaP 78) and AR−/NE+ (LuCaP 93 and LuCaP 145.1) PDX lines. Note the inverse differential enrichment pattern of H3K27ac and H3K27me3 (yellow box) in the upstream regulatory regions of *TACSTD2*, *DLL3*, and *CEACAM5*.

To explore associations between TROP2, PSMA, DLL3, and CEACAM5 expression and somatic genomic alterations, we evaluated logistic regressions and found statistically significantly higher odds of PSMA expression (OR 25; 95% CI 2.4 to 260; p=0.007) in tumors with *AR* amplification but lower odds of PSMA expression in tumors with *RB1* homozygous loss (OR 0.02; 95% CI 0.0 to 0.3; p=0.006) (Figure 3A, **Supplementary Table 3**). We also found lower odds of TROP2 expression in tumors with *RB1* homozygous loss (OR 0; 95% CI 0.0 to 0.02; p<0.001) and in tumors with *PTEN* alterations (OR 0.77; 95% CI 0.77 to 0.77; p<0.001). In independent publicly available transcriptomics and genomics data from 99 mCRPC cases from the StandUp2Cancer West Coast Dream Team (**SU2C-WCDT**), we observed higher odds of CEACAM5 expression in tumors with *PTEN* deletions (OR 4.9; 95% CI 1.3 to 21; p=0.02), lower odds of CEACAM5 expression in tumors with *AR* amplifications (OR 0.2; 95% CI 0.05 to 0.74; p=0.02), and lower odds of DLL3 in tumors with *AR* amplifications (OR 0.06; 95% CI 0.0 to 0.4; p=0.01), and higher odds of TACSTD2 expression with *AR* amplification (OR 13; 95% CI 1.8 to 260; p=0.03) (Figure 3B, **Supplementary Table 4**).

Prior studies have shown that *FOLH1* (PSMA) expression is regulated by an orchestrated interaction between DNA methylation and histone acetylation changes ^20^. To study the epigenetic configuration of TROP2 (encoded by *TACSTD2*), *DLL3*, and *CEACAM5* in tumors with variable levels of target expression, we evaluated previously published whole-genome bisulfite sequencing (**WGBS**) and histone H3 lysine 27 acetyl (**H3K27ac**) and histone H3 lysine 27 tri-methyl (**H3K27me3**) chromatin immunoprecipitation sequencing (**ChIP-seq**) from CRPC patient-derived xenograft (**PDX**) models. We observed that in AR+/NE− PDX lines the *TACSTD2* locus was enriched for H3K27ac marks, consistent with an actively transcribed gene locus (Figure 3C). AR−/NE+ tumors, however, showed gain of the repressive polycomb mark H3K27me3. No consistent DNA methylation changes associated with TACSTD2 were observed (**Supplementary Figure 3**). We further investigated the chromatin patterns at the *DLL3* and *CEACAM5* locus in AR+/NE− and AR−/NE+ tumors and observed H3K27ac enrichment in AR−/NE+ lines. DLL3- and CEACAM5-negative tumors were characterized by enrichment for H3K27me3. Collectively, these data demonstrate that distinct chromatin states are associated with *TROP2*, *DLL3*, and *CEACAM5* expression.

## DISCUSSION

Targeting cell-surface proteins has opened novel avenues for cancer therapy ^12–17^. In advanced metastatic PC, PSMA-directed agents have demonstrated encouraging clinical activity, which culminated in the recent approval of PSMA-directed radioligand therapy 177-Lu-PSMA-617 ^39,40^. However, a notable fraction of mCRPC tumors exhibit insufficient levels of PSMA expression for effective targeting ^41^. Furthermore, heterogeneity in expression that may not be detected on molecular imaging can drive treatment resistance. While experimental approaches to augment PSMA expression are being explored ^20,42^, it is crucial to investigate alternative cell-surface antigens to overcome primary or secondary resistance to PSMA-directed therapies and optimize therapeutic outcomes.

An additional challenge in the treatment of CRPC is the presence of molecular subtypes, which show distinct phenotypic and expression differences ^5^. Importantly, the expression patterns of cell surface proteins vary across these subtypes; for example, PSMA is rarely found in NEPC ^20^, and even within a subtype, there can be substantial heterogeneity in cell surface protein expression ^20,22^.

Pan-cancer analyses have determined that cell surface proteins are being expressed in a lineage-independent manner across multiple tumor types. TROP2 is one such protein that has been shown to be present in multiple epithelial-derived tumors ^34–36^. Sacituzumab govitecan and other TROP2-directed ADCs, including datopotamab deruxtecan, are currently in clinical development ^43^. Sacituzumab govitecan, which has already been approved for triple-negative breast cancer and urothelial carcinoma, has also displayed activity across multiple tumor types and is presently under evaluation in mCRPC (NCT03725761) ^27^.

Given the lack of detailed TROP2 protein expression data in CRPC, we determined TROP2 levels in 52 patients of the UW rapid autopsy cohort. Our analyses revealed that TROP2 protein expression is present in 88% of cases, with 34/52 patients (65%) showing TROP2 expression in all metastatic sites. Prior studies have suggested that TROP2 expression induces a neuroendocrine phenotype and that TROP2 is enriched in NEPC ^44^. However, subsequent *in silico* analyses have demonstrated low TACSTD2 (which encodes for TROP2) transcript levels in NEPC ^20,27^. Similarly, our data show that TROP2 expression is absent in most NEPC (AR−/NE+) tumors. Collectively, TROP2 expression does not appear to be associated with the prognostically poor neuroendocrine subtype, and therefore, TROP2 targeting approaches are likely not effective in NEPC. Notably, compared to PSMA, which we previously analyzed in the same set of tissues ^20^, TROP2 demonstrated more robust and uniform reactivity in most other CRPC tumors. Of particular interest is the high expression of TROP2 in AR−/NE− tumors, a molecular tumor subtype for which there are presently only limited specific therapies ^6^.

Clinically, NEPC represents a major challenge and novel therapies for this aggressive variant of CRPC are needed ^3,10,11^. DLL3 is an inhibitory ligand of the Notch signaling pathway, which has been found to be expressed on the surface of a variety of different neuroendocrine neoplasms, including NEPC ^24,25,45^. Although some early clinical trials with DLL3 ADCs (Rova-T and SC-002) were impeded by systemic toxicities due to payload conjugation concerns, recent studies using bispecific T-cell engagers (such as tarlatamab, BI 764532, and HPN328) have demonstrated encouraging early results ^23,24,46–48^. This expanding spectrum of targeting agents make DLL3 a very interesting and potentially relevant target in NEPC. Our protein expression data corroborated that DLL3 is primarily expressed in AR−/NE+ (NEPC) tumors. Notably, when considering all tumors, DLL3 positivity was limited, but 83% (69/83) of NEPC tumors showed protein expression, while no AR+/NE− tumors exhibiting positivity, indicating that DLL3 is a sensitive and specific marker for NEPC. This information is relevant for the development of DLL3 targeting agents for NEPC imaging.

In addition to DLL3, CEACAM5 has been shown to be expressed at high levels in NEPC ^26^. Although CEACAM5 expression can also be found in gastrointestinal, genitourinary, breast and lung cancers, a recent unbiased surface profiling effort showed a strong enrichment of CEACAM5 expression in NEPC and subsequent *in vivo* models demonstrated activity of a CEACAM5 ADC in NEPC PDX models ^26,30^. While our study confirmed the expression of CEACAM5 in NEPC, we also noted expression in AR−/NE− tumors. Of note, 4/52 (8%) patients showed no expression of TROP2, CEACAM5, DLL3 and PSMA.

Our somatic genomic association studies showed that lower levels of TROP2 and PSMA were present in tumors with homozygous *RB1* loss, whereas higher levels were seen in tumors with *AR* amplification. Conversely, high DLL3 expression was seen in *RB1* deleted cases. While these data present intriguing novel insights between the expression of TROP2, DLL3 and PSMA with common genomic alterations in CRPC, it is important to note that these associations are also tightly associated with tumor phenotype (i.e., *AR* amplification is seen in AR+/NE− tumors, whereas *RB1* loss is enriched in NEPC). Therefore, it is challenging to untangle the genomic alteration from broader cellular state shifts that contribute to differential expression patterns ^8,11,49^.

DLL3 and CEACAM5 have been shown to be regulated by the neuronal transcription factor ASCL1 ^25,26^. Here, we further determined the epigenetic context of these gene loci in tumors with high and low DLL3 and CEACAM5 expression. We observe that the repressive polycomb mark H3K27me3 shows strong enrichment at transcriptional start sites and gene bodies of both genes in PDX tumors with low DLL3 and CEACAM5 expression. Similarly, we show that PDX lines that lack TROP2 expression also showed enrichment for H3K27me3. This contrasts with our prior findings demonstrating that DNA methylation alterations, rather than polycomb marks, are associated with PSMA repression. Thus, polycomb repressive marks, which are established by Enhancer of zeste homolog 2 (**EZH2**), are likely an important epigenetic determinant of TROP2, DLL3 and CEACAM5 expression. It will therefore be important to test in future studies if EZH2 inhibitors, which are currently in clinical development for prostate cancer, can be used to pharmacologically enhance the expression of these cell surface antigens and, therefore, increase tumor targeting.

It is essential to consider several limitations of our study. First, this autopsy-based, single-institution study included only patients with extensive pretreatment. Thus, it remains to be established how our findings would apply to patients in earlier stages of the disease, including castration-sensitive disease. Second, the use of tissue microarray sampling may not entirely capture the intra-tumoral heterogeneity of individual lesions. Additionally, pre-analytical variables must be taken into account, particularly when evaluating bone lesions, as with all studies using formalin-fixed, paraffin-embedded tissues. Despite this potential limitation, it’s worth noting that we did not observe a trend towards lower expression in bone metastasis.

In summary, we have investigated the expression of clinically relevant cell surface targets in mCRPC, providing the most comprehensive tissue-based assessment of TROP2, DLL3, and CEACAM5 in CRPC to date. Our findings highlight the molecular subtype-specific expression of these proteins and provide crucial insights for the future clinical development of these drug targets.

## MATERIALS AND METHODS

### Human tissue samples

This study was approved by the Institutional Review Board of the University of Washington (protocol no. 2341). Formalin-fixed, paraffin-embedded tissues from 52 patients were used to construct tissue microarrays as described previously ^20^.

### Immunohistochemical staining

Slides were deparaffinized and steamed for 45 min in Target Retrieval Solution (Dako Cat. S169984–2). Primary antibodies and dilutions used were as follows: TROP2 (Abcam, ab214488, 1:200), CEACAM5 (Agilent, M7072, 1:20), and PSMA (Agilent, M3620, 1:20). PV Poly-HRP Anti-Mouse IgG (Leica Microsystems Cat. PV6114) or Anti-Rabbit IgG (Leica Microsystems Cat. PV6119) was used as secondary antibody. Further signal amplification was done for CEACAM5 immunostains by using the Biotin XX Tyramide SuperBoost kit (Life Tech Cat. B40931). DLL3 staining was carried out on a Roche Benchmark Ultra instrument (Roche) using DLL3 (Ventana, SP347, 790–7016, 1μg/ml) and the CC1 module. DAB was used as the chromogen and counterstaining was done with hematoxylin and slides were digitized on a Ventana DP 200 Slide Scanner (Roche). Immunoreactivity was scored in a blinded manner by two pathologists (M. P. R., E. S.), whereby the staining level (“0” for no brown color, “1” for faint and fine brown chromogen deposition, and “2” for prominent chromogen deposition) was multiplied by the percentage of cells at each staining level, resulting in a total H-score with a range of 0–200. Note that PSMA and CEACAM5 expression in this cohort were detailed previously ^20^.

### Genomic and epigenomic studies

Somatic alterations of the University of Washington rapid autopsy samples ^5,6,38,50,51^ and genomics calls from the SU2C-WCDT were derived from published sources ^52,53^. ChIP-seq and whole genome bisulfite sequencing data were published previously and analyzed as described previously ^20,54,55^.

### Statistics

Mean H-scores for each cell surface antigen were estimated using linear mixed models with fixed effects for anatomical site and random effects for patients to account for repeated sampling. Associations between expression (dichotomized FPKM) and genomic mutations were evaluated using logistic regressions with random effects for patients to account for repeated sampling. Intra-tumoral and inter-tumoral heterogeneity were estimated by bootstrap random sampling of 1000 pairs of tissue samples from the same tumor block or from the same patient and evaluating whether H-scores were both above or both below a pre-specified threshold of ≥20. Bias-corrected and accelerated 95% confidence limits used the R package Bootstrap ^50^. In all analyses, a p-value <0.05 was considered statistically significant.
